# Adolescent Exposure to the Synthetic Cannabinoid WIN 55212-2 Modifies Cocaine Withdrawal Symptoms in Adult Mice

**DOI:** 10.3390/ijms18061326

**Published:** 2017-06-21

**Authors:** María A. Aguilar, Juan Carlos Ledesma, Marta Rodríguez-Arias, Carles Penalva, Carmen Manzanedo, José Miñarro, M. Carmen Arenas

**Affiliations:** Departamento de Psicobiología, Facultad de Psicología, Universitat de València, Avda. Blasco Ibáñez 21, 46010 Valencia, Spain; marta.rodriguez@uv.es (M.R.-A.); carlespenalva@gmail.com (C.P.); carmen.manzanedo@uv.es (C.M.); jose.minarro@uv.es (J.M.); carmen.arenas@uv.es (M.C.A.)

**Keywords:** adolescence, cannabis, WIN 55212-2, cocaine withdrawal, mice

## Abstract

Chronic cannabinoid consumption is an increasingly common behavior among teenagers and has been shown to cause long-lasting neurobehavioral alterations. Besides, it has been demonstrated that cocaine addiction in adulthood is highly correlated with cannabis abuse during adolescence. Cocaine consumption and subsequent abstinence from it can cause psychiatric symptoms, such as psychosis, cognitive impairment, anxiety, and depression. The aim of the present research was to study the consequences of adolescent exposure to cannabis on the psychiatric-like effects promoted by cocaine withdrawal in adult mice. We pre-treated juvenile mice with the cannabinoid CB1 receptor agonist WIN 55212-2 (WIN) and then subjected them to a chronic cocaine treatment during adulthood. Following these treatments, animals were tested under cocaine withdrawal in the following paradigms: pre-pulse inhibition, object recognition, elevated plus maze, and tail suspension. The long-term psychotic-like actions induced by WIN were not modified after cocaine cessation. Moreover, the memory impairments induced by cocaine withdrawal were not altered by previous adolescent WIN intake. However, WIN pre-treatment prevented the anxiogenic effects observed after cocaine abstinence, and led to greater depressive-like symptoms following cocaine removal in adulthood. This study is the first to show the long-lasting behavioral consequences of juvenile exposure to WIN on cocaine withdrawal in adult mice.

## 1. Introduction

Products of the hemp plant *Cannabis sativa*—marijuana and hashish are among the illicit substances most commonly used by adolescents and young adults worldwide, and are the world’s third most popular recreational drug after ethanol (EtOH) and tobacco [1]. Most cannabis users start in their mid-to-late teens, coinciding with major neuronal changes in the central nervous system [1]. The main psychoactive component of the Cannabis sativa plant and its derivatives is Δ9-tetrahydrocannabinol (THC), which acts primarily via specific endogenous cannabinoid 1 (CB1) receptors in the brain [2,3]. In recent years, synthetic cannabinoids have become very popular. Since 2008, over 160 synthetic cannabinoids have been detected in a range of different products that are sold as “legal” replacements for cannabis although they are considerably more toxic than marijuana and hashish [4]. Synthetic cannabinoids are also several times more potent than THC in activating cannabinoid receptors in the brain [5]. The endocannabinoid system is densely distributed in regions involved in the processing of emotional inputs, rewarding stimuli, habit formation, and higher cognitive functions, and represents a common neurobiological substrate for the addictive properties of different drugs of abuse via CB1 receptors [6,7].

Cannabinoid consumption, especially during pubertal development, induces long-lasting neurobehavioral alterations, psychiatric diseases (e.g., schizophrenia, depression, and anxiety disorders) and cognitive impairments in adulthood [8,9,10]. In experimental rodents, it has been demonstrated that chronic exposure to cannabinoid agonists during adolescence, but not adulthood, leads to neurobehavioral alterations in adult animals (for a review, see [11,12,13]). In particular, the administration of the synthetic CB1 agonist WIN 55212-2 (WIN) to rats and mice during the adolescent period leads to long-lasting deficits in different behavioral paradigms, such as fear conditioning [14,15], progressive ratio food self-administration [16] and pre-pulse inhibition of the acoustic startle response [14,16,17]. Other authors have reported that WIN-exposed rats show only increased acoustic startle latency, but not overt deficits in sensoriomotor gating or modifications of social interaction suggesting that exposure to WIN during adolescence does not lead to psychotic-like behavior [18]. Conversely, other authors have demonstrated that mice treated with WIN show an impairment in an attentional set-shifting task, supporting the notion that exposure to cannabinoids during adolescence may represent a risk factor for developing schizophrenia-like signs at adulthood [19]. Chronic pubertal WIN treatment also stimulates locomotion [17], increases novel-object exploration [18], induces depression-like behavior in the forced swim and sucrose preference tests [20] and alters anxiety-like behavior [17,20]. However, the effects of WIN on anxiety are in function of the paradigm used to evaluate anxiety. WIN reduces anxiety levels in the elevated plus-maze [17], but it induces anxiety-like behavior in the novelty-suppressed feeding test [20]. Moreover, treatment with WIN during adolescence impairs water maze performance [15] and object recognition memory [16], suppresses cortical oscillations (patterns of neural network activity implicated in cognitive processing) [21], and induces long-term depression in the nucleus accumbens [22]. Some studies have observed a recovery of cannabinoid-induced cognitive deficits. Chronic WIN administration during late adolescence induces an enduring impairment of hippocampal dependent short-term memory measured in the object location task, but the deficits in the water maze and the object recognition tasks as well as the impairment of long-term potentiation in the ventral subiculum-nucleus accumbens pathway are temporary [23]. A recent study confirms that the administration of WIN during adolescence acutely impairs short-term memory, but has no effect on working memory performance in adulthood [24]. In addition, this study reports an improvement in the performance of a delay-match-to-sample working memory task in adult rats self-administering WIN during adolescence [24]. Biochemical and structural brain alterations have also been reported after adolescent treatment with WIN, such as reductions in endocannabinoid signaling and mGluR5 in the hippocampus [14], higher hippocampal anandamide levels [15], an increased turnover of striatal dopamine (but with unchanged dopamine levels) [18], higher number of active dopaminergic neurons in the ventral tegmental area [19], serotonergic hypoactivity in the dorsal raphe and noradrenergic hyperactivity in the locus coeruleus [20], impaired maturation of prefrontal GABAergic transmission [25], loss of dendritic spine density in the dentate gyrus [26], and frontostriatal gliogenesis [18].

Interestingly, an important long-lasting consequence of cannabis use during adolescence is a heightened risk of developing other addictive diseases in adulthood. It has been reported that early onset and higher frequency of cannabis consumption is a reliable predictor of later problematic abuse of other illicit drugs in adulthood [27]. This is known as the “gateway hypothesis”, which states that a causal relationship exists between early exposure to drugs of abuse and the subsequent abuse of other addictive substances later in life [11,28,29]. In animal models, the exposure of WIN during adolescence also affects the response to drugs of abuse at adulthood. For example, rats treated with WIN during adolescence showed a long-term reduced activation of midbrain dopaminergic neurons in response to morphine, cocaine and amphetamine [30]. However, other studies reported an increase of amphetamine-induced psychomotor sensitization [19], while others have not found an influence of adolescent WIN exposure on neurochemical and behavioural effects of amphetamine [31]. Similarly, previous studies of our laboratory with adolescent mice demonstrated that WIN pre-exposure strengthens the rewarding properties of 3,4 methylenedioxymetamphetamine (MDMA) and favors reinstatement of the craving for this drug [32]. The administration of WIN during adolescence can even induce transgenerational effects since offspring of female rats treated with WIN during adolescence (but not during pregnancy) exhibited greater sensitivity to the conditioned rewarding effects of morphine [33] as well as an increased sensitization to morphine-induced hyperactivity and higher levels of mu opioid receptor in the nucleus accumbens [34]. Regarding cocaine, the most widely used illicit drug in developed countries after cannabinoids, it has been demonstrated that the development of addiction to cocaine is strongly correlated with an early history of cannabis abuse in adolescence [35,36,37], which is also in accordance with the gateway hypothesis. In animal models, WIN administration during adolescence increases the rewarding effects of cocaine in mice [38]. Cocaine addiction is a chronic behavioral disturbance characterized by compulsive intake and relapse after short periods of abstinence [39]. Abuse of this substance and its subsequent withdrawal can cause psychiatric symptoms, among which psychosis, cognitive impairment, anxiety, and depression prevail [40,41,42,43,44]. However, although a link has been said to exist between cannabis consumption earlier in life and later development of cocaine addiction, there are no studies that have examined the behavioral outcomes of this pattern of drug ingestion in animals.

Therefore, the aim of the present study was to evaluate the effect of adolescent exposure to WIN on the psychiatric-like actions evoked by cocaine withdrawal. For this purpose, we pre-treated juvenile mice chronically with WIN and then submitted them to a chronic cocaine treatment during adulthood. Given that it has been established that periadolescent cannabis abuse has long-lasting psychiatric consequences, we tested the effect of these treatments on psychotic-, cognitive-, anxiety-, and depressive-like symptoms, in adult mice following cocaine withdrawal. Locomotor activity of mice was also tested. The results derived from the present research highlight the possible increased risk of developing some psychopathologies among subjects with an earlier history of cannabis abuse and cocaine consumption in adulthood.

## 2. Results

### 2.1. Open Field

**Figure 1:**
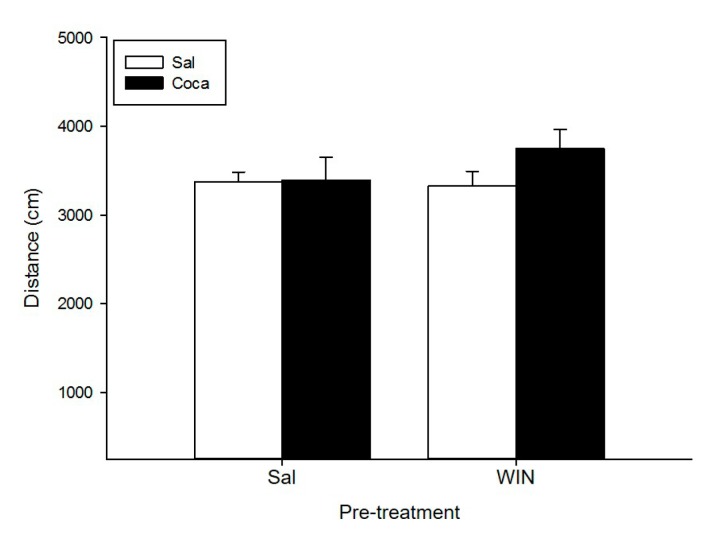
Effect of WIN 55212-2 administration during adolescence on locomotor activity after cocaine withdrawal in adulthood. Over post-natal day (PND) 34–47 (adolescence), mice (*n* = 10 per group) were pre-treated with physiological saline (Sal) or WIN 55212-2 (WIN 0.5 mg/kg given once daily). In adulthood (21 days later) they were treated with Sal or cocaine (Coca) in three daily injections separated by a 60-min interval according to the ensuing regime: 5 mg/kg on PND 68 and 69, 15 mg/kg from PND 70 to 72, a two-day abstinence period, and 25 mg/kg from PND 75 to 79. Animals were tested on PND 80. Bars depict mean ± standard error mean (SEM) locomotor activity (distance travelled in cm/10 min) for all drug treatment groups.

None of our treatments had an effect on the locomotor activity of mice (Figure 1). The ANOVA did not show any significant effects of the Pre-treatment, the Treatment, or the Interaction Treatment X Pre-treatment.

### 2.2. Pre-Pulse Inhibition

**Figure 2:**
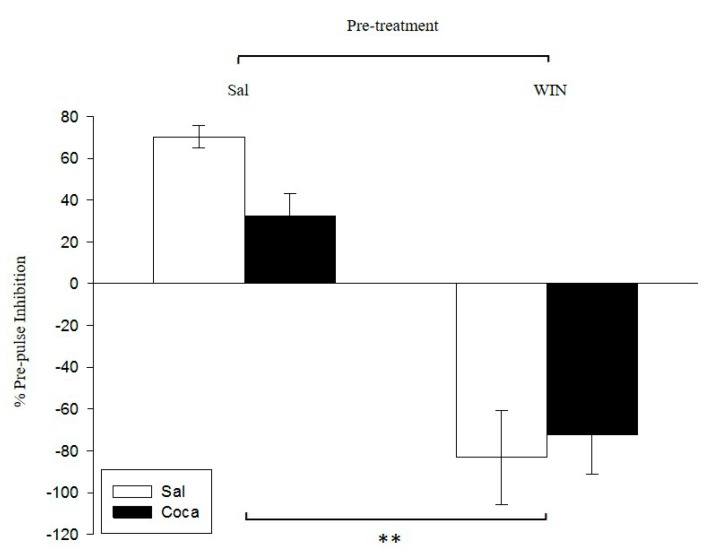
Effect of WIN 55212-2 administration during adolescence on pre-pulse inhibition after cocaine withdrawal in adulthood. Over PND 34–47 (adolescence), mice (*n* = 10 per group) were pre-treated with physiological saline (Sal) or WIN 55212-2 (WIN 0.5 mg/kg given once daily). In adulthood (21 days later) they were treated with Sal or cocaine (Coca) in three daily injections separated by a 60-min interval according to the ensuing regime: 5 mg/kg on PND 68 and 69, 15 mg/kg from PND 70 to 72, a two-day abstinence period, and 25 mg/kg from PND 75 to 79. Animals were tested on PND 81. Bars depict mean ± SEM of percentage of the pre-pulse inhibition response for trials with a pre-pulse of 75 dB with inter-stimulus intervals of 100 ms, and a pulse of 120 dB for all groups (** *p* < 0.01 significant differences between Sal and WIN pre-treated mice).

The ANOVA performed to analyze the pre-pulse inhibition (Figure 2) revealed a significant effect of pre-treatment (*F*(1,36) = 66.81, *p* < 0.01), thereby indicating that the % of PPI of the WIN pre-treated mice (i.e., WIN–Sal and WIN–Coca) was significantly lower than that of the Sal pre-treated groups (Sal–Sal and Sal–Coca). The variable treatment and the interaction treatment X pre-treatment were not significant.

### 2.3. Object Recognition

**Figure 3:**
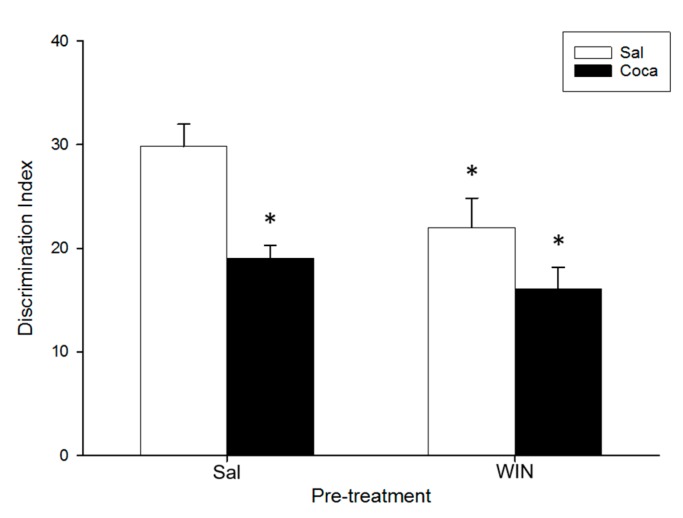
Effect of WIN 55212-2 administration during adolescence on novel object recognition after cocaine withdrawal in adulthood. Over PND 34–47 (adolescence) mice (*n* = 10 per group) were pre-treated with Sal or WIN (0.5 mg/kg given once daily). In adulthood (21 days later) they were treated with Sal or Coca in three daily injections separated by a 60-min interval according to the ensuing regime: 5 mg/kg on PND 68 and 69, 15 mg/kg from PND 70 to 72, a two-day abstinence period, and 25 mg/kg from PND 75 to 79. Animals were tested on PND 82. Bars depict mean ± SEM of the Discrimination Index (DI) for all groups (* *p* < 0.05 significantly different from the Sal–Sal group).

The ANOVA of the data for the object recognition (Figure 3) indicated a significant effect of Pre-treatment (*F*(1,36) = 6.29, *p* < 0.05), and Treatment (*F*(1,36) = 15.11, *p* < 0.01). The Tukey honest significant difference (HSD) post hoc test showed that the Discrimination Index (DI) of the Sal–Coca, the WIN–Sal, and the WIN-Coca groups was diminished in comparison with the Sal–Sal group (*p* < 0.05). No significant differences were encountered between the WIN–Sal and the WIN–Coca groups (*p* > 0.05).

### 2.4. Elevated Plus Maze

Table 1 presents the results obtained in the elevated plus maze (EPM). The ANOVA for both the time that the animals spent in the open arms (Time OA), the percentage of time in the open arms (% time OA), and the percentage of entries in the open arms that they performed (% entries OA) revealed a significant effect of Pre-treatment ((*F*(1,36) = 23.95, *p* < 0.01), (*F*(1,36) = 23.95, *p* < 0.01), and (*F*(1,36) = 7.79, *p* < 0.01), respectively), Treatment ((*F*(1,36) = 29.75, *p* < 0.01), (*F*(1,36) = 29.75, *p* < 0.01), and (*F*(1,36) = 28.14, *p* < 0.01), respectively), and Interaction ((*F*(1,36) = 33.16, *p* < 0.01), (*F*(1,36) = 33.16, *p* < 0.01), and (*F*(1,36) = 8.46, *p* < 0.05), respectively). The post hoc test showed that Time OA, % time OA, and % entries OA were significantly lower in the Sal–Coca group than the rest of the groups (*p* < 0.01). For the number of entries that they carry out in the open arms (OA entries), the ANOVA found a significant effect of Treatment (*F*(1,36) = 15.66, *p* < 0.01) and Interaction (*F*(1,36) = 8.85, *p* < 0.01). Pairwise comparisons indicated that the Sal–Coca group carried out fewer OA entries than the other groups (*p* < 0.05). For the time spent in the closed arms of the maze (Time CA), the ANOVA exhibited a statistical effect of Pre-treatment (*F*(1,36) = 11.94, *p <* 0.01), Treatment (*F*(1,36) = 14.84, *p* < 0.01), and Interaction (*F*(1,36) = 4.04, *p* < 0.05). The post hoc test revealed that the Sal–Coca group spent more Time CA than the other groups (*p* < 0.01). For the number of entries in the closed arms (CA entries), the ANOVA reflected an effect of Pre-treatment and Treatment (*F*(1,36) = 8.84, *p* < 0.01) and (*F*(1,36) = 6.94, *p* < 0.05), respectively. The Tukey HSD test indicated that the Sal–Coca group performed more CA entries than the rest (*p* < 0.05). No significant differences were obtained between groups with respect to the total entries in both arms (Total entries) and the time remained in the center (Time center).

### 2.5. Tail Suspension Test

**Figure 4:**
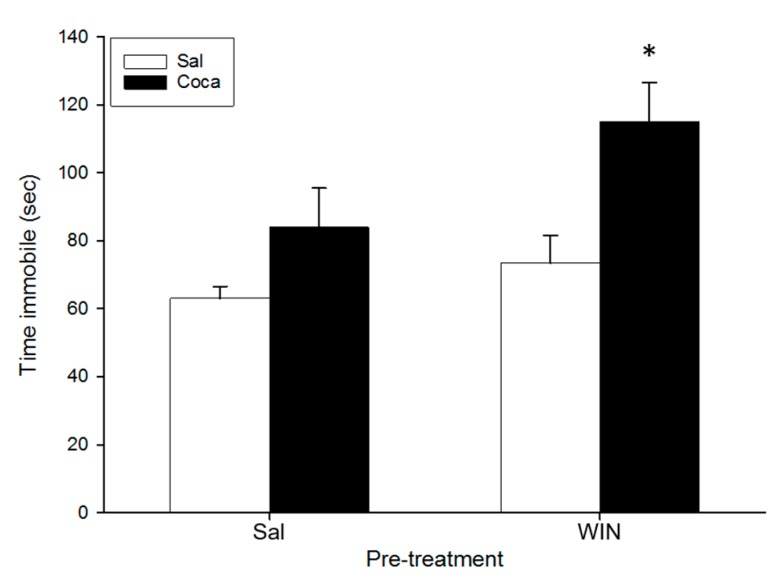
Effect of WIN 55212-2 administration during adolescence on the tail suspension test after cocaine withdrawal in adulthood. Over PND 34–47 (adolescence) mice (*n* = 10 per group) were pre-treated with Sal or WIN (0.5 mg/kg given once daily). In adulthood (21 days later) they were treated with Sal or Coca in three daily injections separated by a 60-min interval according to the ensuing regime: 5 mg/kg on PND 68 and 69, 15 mg/kg from PND 70 to 72, a two-day abstinence period, and 25 mg/kg from PND 75 to 79. Animals were tested on PND 83. Bars depict mean ± SEM of the time (s) during which the mice remained immobile in the tail suspension test (* *p* < 0.05 significantly different from the rest).

For the tail suspension test (Figure 4), the ANOVA indicated a significant effect of Pre-treatment (*F*(1,36) = 5.05, *p* < 0.05) and Treatment (*F*(1,36) = 11.5, *p* < 0.01). The Tukey HSD post hoc test indicated that the time spent immobile was higher in the WIN-Coca group in comparison to the rest (*p* < 0.05).

## 3. Discussion

The results reported here represent a first approach towards assessing the long-lasting consequences of juvenile exposure to WIN on cocaine withdrawal in adult mice. We used a murine model of pubertal subchronic cannabis intake followed by a chronic cocaine binge regime in adulthood, thereby imitating a pattern seen in many human polydrug users [28,45]. A limitation of this study is the use of the synthetic CB1 cannabinoid agonist WIN instead of THC, which is the main psychoactive component of cannabis. However, there are several reasons for the use of WIN instead of THC. Firstly, in previous works of our laboratory on the influence of adolescent cannabinoid exposure on the rewarding effects of other drugs of abuse, we administered WIN following similar procedures (i.e., drug dose, treatment regime, strain and age of the subjects; see [32,38,46,47,48] for examples), thus giving us the possibility to compare the results obtained between the different studies. Secondly, the vehicle used to dissolve THC (a mixture of ethanol plus another solvent such as Cremophor) can induce behavioral side effects per se (unpublished data), while that of WIN (physiological saline with a low concentration (<3%) of Tween 20) is innocuous for mice [49]. Thirdly, WIN possesses higher affinity for cannabinoid CB1 and CB2 receptors than THC [5] and has been widely used to study the implication of the endocannabinoid system in different behaviors in rodents as well as in animal models of cannabinoid dependence [14,15,16,17,18,19,20,50]. In particular, WIN is self-administered by rodents [24,50] and induces cross-tolerance and cross-discrimination with THC [50]. Thus, any effects of THC on behavior are likely to be replicated by WIN. Therefore, although the effects of WIN would be more comparable to those of the abused synthetic cannabinoids (K2, Spice, etc.) than to those of cannabis [51], the use of WIN can contribute to clarifying the effects of adolescent cannabinoid exposure on the behavioral alterations induced by cocaine abstinence.

We have found that the long-term psychotic-like effects induced by WIN administration are not altered by cocaine abstinence. Furthermore, the short-term memory deficits evoked by cocaine are not modified after earlier administration of WIN, which itself induces a long-lasting memory impairment. Interestingly, we have shown that adolescent cannabinoid treatment prevents the anxiogenic effects observed after cocaine abstinence in adult mice. Finally, we observed that the combination of these treatments enhanced the risk of suffering from depressive-like symptoms. Additionally, in order to rule out any confounding factor in the rest of the behavioral results, we have confirmed that none of our pharmacological manipulations affect the spontaneous locomotor activity.

In the PPI paradigm, we observed that adult mice treated with WIN during adolescence showed an increase in sensorimotor gating. This outcome is in line with earlier reports, which have demonstrated that WIN administration only induces a long-term impairment of the PPI response when is given during the adolescent period [14,16,17]. Hence, it seems that cannabinoid consumption early in life raises the risk of suffering from schizophrenia, as was observed in human beings [52]. Conversely, we found no significant effects of cocaine abstinence in adult mice treated with saline during adolescence. These results, in the same line as previous studies with rats [53,54] and mice [55], suggest that cocaine withdrawal by itself does not induce psychotic-like actions. As can be seen in Figure 2, while the administration of WIN during adolescence clearly impairs PPI when the mice become adults (WIN-Sal group), there is a trend towards a reduction in the PPI of mice treated only with cocaine in adulthood when they are in abstinence (Sal-Coca group). Therefore, it could be plausible to hypothesize that the administration of both drugs will induce synergistic impairing effects on PPI, but it does not seem to be the case. Mice treated with the combination of WIN during adolescence and cocaine in adulthood showed a disruption of PPI similar to that observed in mice treated only with the cannabinoid agonist. This incongruity could be explained by a ceiling effect on the disruption of PPI induced by WIN exposure. In an earlier study, we demonstrated that exposure to an EtOH binge-drinking procedure during adolescence, which is not able to alter the PPI by itself, impairs the startle reflex in mice exposed to the same cocaine withdrawal as that of the current study [55]. This supports the notion that previous drug exposure during the adolescent period may enhance the putative psychotic-like actions of cocaine in mice. Further studies may be carried out to test this possibility, for example by modifying the PPI parameters such as the intensity and the duration of the pre-pulse, or by using different doses and timings of both WIN and cocaine delivery.

In the OR test, adolescent WIN pre-treatment disrupted short-term memory in adult mice treated with saline, in agreement with previous reports showing that chronic pubertal cannabinoid administration impairs the execution of this task in adult rodents [16,56,57]. Nevertheless, a more recent study shows that chronic adolescent administration of THC does not alter memory of adult rats [58]. A possible explanation for this discrepancy could be due to methodological differences between these studies. In relation to the effects of cocaine abstinence, we have found that mice treated with saline during adolescence undergoing withdrawal from cocaine in adulthood performed the OR task more poorly than control subjects (Sal–Sal group), that is in agreement with previous studies in rodents [55,59,60]. Similarly, mice pre-treated with WIN during adolescence undergoing cocaine withdrawal performed the OR task more poorly than the control subjects (Sal-Sal group). However, given that there were no differences in the DI between the WIN-Coca group and the WIN-Sal or Sal-Coca groups, we cannot conclude that WIN administration interacts with cocaine to enhance the cognitive deficits that both drugs produced by themselves. Notwithstanding, this is the first study to assess the effects of the possible interaction between the two treatments on cognitive function, and future research is needed to evaluate its effects on memory more thoroughly (for example by using schedules of WIN and cocaine administration that did not themselves impair recognition memory).

In the EPM, the means of the total entries and the time in center did not vary among the groups, thereby confirming that, as occurred in the OF, the locomotor activity of mice was not altered after our treatments. Moreover, we found that adolescent WIN administration did not affect the anxiety scores of adult mice. To date, the few studies evaluating the long-lasting effects of chronic cannabinoid administration on the behavior of adult rodents in the EPM have been performed in rats and have obtained controversial results. Pubertal WIN exposure reduced the anxiety of adult rats in one study [17] while adolescent THC treatment elicited anxiety-like effects in adult rats in another study [61]. Given that WIN is a synthetic derivative from THC, it may be possible that it does not act in the brain in exactly the same way as THC, thus leading to divergent results. Further research is needed to determine the long-lasting effects of cannabis and its derivatives on anxiety. With respect to the effects of cocaine abstinence, we demonstrated that cessation of cocaine administration in adult mice treated with saline during adolescence evoked anxiety-like behaviors, in agreement with earlier studies in rats [41,62,63,64,65,66] and mice [55]. However, a very interesting result of the present study is that the anxiety values displayed by the animals pre-treated with WIN and later with cocaine did not differ from those of control mice. It could be interpreted from this that juvenile exposure to WIN alleviates the anxiety withdrawal symptoms associated with cocaine abstinence in adult mice. Since high anxiety is one of the factors that contribute to relapse, because many cocaine-abstinent addicts consume the drug again to alleviate the anxiety induced by the withdrawal syndrome [67,68], one can speculate that cannabinoid consumption in adolescence protects against cocaine relapse after its abstinence in adulthood. However, an alternative interpretation might be that prior exposure to WIN, by reducing the anxiogenic effects induced by cocaine cessation, maintains cycles of consumption and abstinence because subjects experience the aversive consequences of the abstinence syndrome to a lesser degree. Similar to the observed in the present research, we have previously proven that mice exposed to an EtOH-binge drinking procedure during adolescence do not exhibit anxiety-like effects after cocaine removal in adulthood [55]. This suggests that adolescent exposure to drugs such as alcohol or cannabis could be a factor that modulates the anxiety related with cocaine withdrawal. Hence, taking into account that it has been reported that periadolescent abuse of both alcohol and cannabis is a high predictor of later development of cocaine addiction [27,29,35], it is possible that the alleviation of anxiety that results as a consequence of the earlier intake of these drugs may play an important role in the acquisition and maintenance of addiction to cocaine. This could be an appealing forthcoming line of research to be explored.

In the TST, depressive-like effects were not observed in adult mice treated with the cannabinoid agonist during adolescence. Studies evaluating the long-term consequences of adolescent cannabis exposure on depression-like behavior are scarce but a previous study showed that chronic WIN administration during adolescence led to a depression-like profile in the forced swim and sucrose preference tests in adult rats [20]. More research is therefore needed to determine the putative depressive-like properties of early cannabinoid exposure. Cocaine abstinence did not significantly increase immobility time with respect to the control group (Sal-Sal), in agreement with our previous study [55]. Some researchers have also found that abstinence from chronic cocaine did not induce any depression-like actions in rats using the sucrose preference or the forced swim tests [64], although in other studies cocaine withdrawal is accompanied by depression-like symptoms [42,69,70]. The tendency of cocaine to increase immobility scores was potentiated in abstinent mice pretreated with WIN during adolescence, since the WIN-Coca group showed a greater immobility time than the Sal-Coca group (and also than the WIN-Sal and Sal-Sal groups). Thus, it seems that a history of abuse of WIN during adolescence and cocaine discontinuation after its chronic intake at an adult age could predispose the subjects to suffer from depressive-like symptoms. A very similar result was obtained from a previous report from our laboratory, in which we demonstrated that adult mice under cocaine abstinence also exhibited a depressive profile in the TST, but only when being previously exposed to an EtOH binge-drinking treatment during the adolescent period [55]. Therefore, it seems that an earlier history of adolescent consumption of cannabis or EtOH might facilitate the development of later depressive disorders after cocaine abuse during adulthood.

## 4. Materials and Methods

### 4.1. Animals

Male mice of the OF1 strain purchased from Charles River (Barcelona, Spain) were used. The subjects were 21 days old upon arrival at the laboratory and were all housed under standard conditions in groups of four, at a constant temperature (21 ± 2 °C) and relative humidity (60%), with a reversed light schedule (white lights on 19:30–7:30). Food and water were provided ad libitum throughout the study. All the procedures involving the mice and their care complied with national, regional, and local laws and regulations, which are in accordance with the Directive 2010/63/EU of the European Parliament and the Council of 22 September 2010 on the protection of animals used for scientific purposes. The protocol was approved by the Ethical Committee for Animal Experiments of the University of Valencia.

### 4.2. Drugs

All drugs were diluted in physiological saline (NaCl 0.9% *w*/*v*; Sal) and injected intraperitoneally (IP). The selective CB1 receptor agonist WIN 55212-2 (WIN), obtained from Tocris Biogen Científica S.L. (Madrid, Spain), was dissolved in Sal mixed with two drops of Tween-80 (Sigma-Aldrich, Madrid, Spain), and dispensed at a dose of 0.5 mg/kg. Cocaine hydrochloride (Coca) was supplied by Laboratorios Alcaliber S.A. (Madrid, Spain) and administered at doses of 5, 15, and 25 mg/kg.

### 4.3. Experimental Design

On postnatal day (PND) 34, corresponding to early adolescence in humans [57], animals were injected with Sal or WIN once a day for 14 consecutive days. This dose of WIN was selected based on previous studies showing that it is able to modulate several behaviors in mice such as morphine- and MDMA-induced conditioned place preference, ingestive behavior, and EtOH consumption, among others [32,38,46,71,72,73]. After WIN pre-treatment, animals were untouched for three weeks, until cocaine administration began on PND 68, an age considered to represent adulthood in mice [56]. The procedure of cocaine administration was a variation of that described by [74], which has been proven to induce long-lasting alterations in rodent behavior [45,74]. In short, cocaine (or Sal administered at the same volume to control animals) was given to mice in ascending doses over a period of 12 days starting on PND 68, when they received three injections per day, separated by a 60 min interval, of either Sal or 5 mg/kg of cocaine (Coca 5) on PND 68 and 69, 15 mg/kg (Coca 15) on PND 70, 71, and 72 (followed by two-day period of abstinence), and 25 mg/kg (Coca 25) on PND 75, 76, 77, 78, and 79. As a result, the following experimental groups were constituted: Sal-Sal: the control group, which received Sal throughout all the experimental phases; Sal-Coca: this group received Sal during the first phase (adolescence) and cocaine during the second (adulthood); WIN-Sal: this group was treated with WIN in adolescence and with Sal in adulthood; WIN-Coca: this group was injected with WIN during adolescence and with cocaine during adulthood.

### 4.4. Behavioral Testing

#### 4.4.1. Open Field

The open field (OF) experiment was performed on PND 80 with the aim of assessing whether our pharmacological manipulations induced any unspecific effect on the normal spontaneous locomotor activity of mice that could have obscured the results obtained in the rest of the experiments. For that purpose, mice (*n* = 10 per group) were introduced individually into locomotor activity chambers consisting of a square arena (30 × 30 × 35 cm^3^) illuminated by a dim white light (40 lx). Horizontal locomotion was recorded as cm travelled in 10 min by a computerized video-tracking system. The movement of the mice inside the OP chamber was registered and transferred automatically to the computer using the software application Ethovision 2.0. (Noldus, Wageningen, The Netherlands).

#### 4.4.2. Pre-Pulse Inhibition

Pre-pulse inhibition (PPI) of the acoustic startle response has been widely used as a measure of sensorimotor gating [75,76,77]. PPI is the normal reduction of the amplitude of the startle reflex in response to an intense startling stimulus (pulse, >100 dB) when this stimulus is shortly preceded by a weaker, non-startling sensory stimulus (pre-pulse, <85 dB). This paradigm has been used to explore the information-processing deficits that typically occur in subjects with psychosis, because human patients suffering from schizophrenia have PPI impairments [78,79]. It is a cross-species translational model that allows some features of schizophrenia to be studied [76,80,81].

We used a PPI apparatus consisting of a Plexiglas tube (28 × 15 × 17 cm^3^) with a platform containing a sensor on its base, so that if the animal moves, the force exerted on the platform is detected. The movements caused by the startle are transduced by an accelerometer and the signal is recorded and digitized by a microcomputer that is also used to present the stimulus and collect the data. The unit is placed in a soundproofed chamber (90 × 55 × 60 cm^3^) that is constantly lit (lamp 10 w) and equipped with two 28-cm speakers located 15 cm from the two sides of the Plexiglas box. These speakers are connected to an amplifier, which in turn is connected to a noise generator that manages the acoustic stimulus and a second noise generator that produces the signal corresponding to the pre-pulse. The apparatus (model CERS41, Computacisponse ciaitor (CERS41) Hardware ón Estimulador Registrador Startle Response) and program that collect the data were purchased from CIBERTEC, S.A. (Madrid, Spain).

The procedure, based on that used by van den Buuse [80], was carried out in two phases. The first was the acclimatization phase (on PND 80), in which mice (*n* = 10 per group) were placed in the animal holder for 5 min with a background noise of 65 dB but no startle stimuli. In the second phase (PND 81), after another 5-min acclimatization period, a series of trials were introduced in a pseudo-random order while the white noise kept playing in the background. The trials consisted of a startle pulse alone (120 dB), a pre-pulse alone (75 dB), and pulses with pre-pulse (pre-pulse of 75 dB with inter-stimulus intervals of 100 ms and a pulse of 120 dB) with an inter-trials interval of 20 s and a duration of 4 ms. The pre-pulse was introduced to verify that it was not acting as a pulse and to confirm that only the 120-dB pulse was acting as the main stimulus to induce the startle response in the animal. PPI was calculated as a percentage score: PPI (%) = 100 − (startle response for pulse with pre-pulse × 100/startle response for pulse alone).

#### 4.4.3. Object Recognition

Object recognition (OR) tests episodic memory in rodents, is similar to methods used in clinical neuropsychology, and is considered a suitable model to investigate the effects of pharmacological manipulation on learning and memory [82]. It has been used as a measure of cognitive dysfunction according to deficits in object-context identification [83]. In the present study, it was performed in an open box (24 × 24 × 15 cm^3^) divided into four equally sized arenas by a sheet of cardboard. A video camera fixed to the ceiling enabled the four arenas to be visualized simultaneously. For the test, two types of objects were used: two small river stones and a small non-toxic plastic toy. This task consists of three phases: habituation, training session (T1), and test session (T2). In the habituation phase (PND 81), mice (*n* = 10 per group) were placed in the center of the empty box and allowed to explore it freely for 2 min. Twenty-four hours later (PND 82), at T1, subjects were introduced for 3 min in the box, which contained the stones each placed in opposite corners. Following this, the mice were returned to their home cage for 1 min (memory retention interval). For T2, we replaced one of the stones with a toy and, after the memory retention interval, the animals were placed once again in the box for another 3 min to evaluate their exploration of the novel object. Object exploration was defined as intentional contact of the mouse's snout or front paws with the object from a distance of 2 cm or less. The following behaviors were scored: sum of the total time (*t*, seconds) spent exploring the two stones at T1, sum of the time spent exploring the stone and the toy at T2, and the difference between the time spent exploring the stone and the toy at T2. The basic measure of memory acquisition was the discrimination index (DI), calculated as: DI = (*t*_novel_ − *t*_familiar_)/(*t*_novel_ + *t*_familiar_) × 100% [84].

#### 4.4.4. Elevated Plus Maze

The elevated plus maze (EPM) paradigm is based on the natural aversion of mice to open elevated areas, and also on the natural spontaneous exploratory behavior they exhibit in novel environments. It measures the extent to which the rodents avoid high open spaces. In our experiment it consisted of two open arms (30 × 5 cm^2^) and two enclosed arms (30 × 5 cm^2^), and the junction of the four arms formed a central platform (5 × 5 cm^2^). The floor of the maze was made of black plexiglas and the walls of the closed arms were made of clear plexiglas. The open arms had a small edge (0.25 cm) to provide the animals with additional grip. The entire apparatus was elevated 45 cm above floor level. The total time spent in the open and closed arms, the number of entries into the open and closed arms, and the percentage of time and entries in the open arms are commonly considered indicators of open space-induced anxiety in mice. The total entries in both arms and the time spent in the center are regarded as locomotor activity scores (for more details, [85,86]).

At the beginning of each trial (PND 82), subjects (*n* = 10) were placed on the central platform of the EPM and were allowed to explore it for 5 min. The behavior displayed by the mice was video recorded and the following measurements were taken into account for the statistical analyses: total time spent in the open arms (Time OA), percentage of time in the OA (% time OA), number of entries into the OA (OA entries), percentage of entries into the OA (% entries OA), total time spent in the closed arms (Time CA), number of entries into the CA (CA entries), total entries, and total time spent on the central platform (Time center).

#### 4.4.5. Tail Suspension

The tail suspension test (TST) measures the behavioral variable of immobility, which is considered to represent despair [87]. It is based on the observation that rodents, after initial escape-oriented movements, develop an immobile posture when placed in an inescapable stressful situation. In the case of the TST, the stressful situation involves the hemodynamic stress of being hung in an uncontrollable fashion by their tail [88]. This has been used as a measure of behavioral depression because, when antidepressant treatments are given prior to the test, the subjects will engage in escape-directed behaviors for longer periods of time than after treatment with the vehicle [88].

Following the protocol described by [89], on PND 83 adhesive tape was used to suspend mice (*n* = 10 per group) by the tail from a hook connected to a strain gauge that recorded their movements during a 6-min test period. The behavior displayed by the mice was video recorded and the parameter considered for the statistical analyses was the total time spent immobile.

The order of the different tests undergone by the mice was selected based on pilot studies conducted in our laboratory that demonstrated the absence of any influence of previous testing on the subsequent test. As the TST is the most stressing, it was performed last. In a previous study [55] we have used a slightly different schedule of behavioral testing obtaining the same results in control mice. A schematic description of the experimental procedure is provided in Table 2.

### 4.5. Statistical Analyses

Data were analyzed by means of two-way ANOVA with two between-subjects variables: pre-treatment (with two levels: Sal and WIN) and treatment (with two levels: Sal and Coca), followed by Tukey’s honest significant difference (HSD), a post hoc test that does not require a significant interaction between factors and is highly conservative against type I error [90]. The α level was set at *p* < 0.05 for all analyses.

## 5. Conclusions

In summary, the current results demonstrate for the first time the long-lasting consequences of juvenile exposure to WIN on cocaine withdrawal in adult mice. In addition, we corroborate the long-term effects of adolescent WIN administration in the disruption of the PPI, and the deleterious effects of both WIN and cocaine on memory retention previously shown by others. The most important item of data derived from our experiments is that we found an interaction between prior WIN consumption during adolescence and later abstinence from cocaine, in anxiety- and depressive-like behaviors. Exposure to WIN during adolescence prevents the anxiety-like effects observed in mice withdrawn from cocaine in the EPM. Moreover, mice treated with WIN during adolescence showed a specific increase in depressive-like symptoms in the TST during cocaine abstinence. Therefore, it seems that adolescent cannabinoid exposure could modulate the vulnerability to suffer from different behavioral disturbances after later cocaine withdrawal. To verify this hypothesis more thoroughly and to strengthen the present results, it would be suitable in the future to carry out these same experiments using the CB1 receptor agonist WIN together with a CB1 antagonist.

Given that pubertal cannabis use followed by later cocaine abuse is an increasingly common pattern of drug consumption in human beings, the results of the present research are particularly relevant, as they provide evidence that this pattern of drug intake could alter some of the behavioral outcomes produced by these drugs when given alone. Moreover, the results of the present work link directly with those of an earlier report, showing that adolescent binge EtOH exposure also modulates cocaine withdrawal symptoms [56]. Therefore, taking into account the high incidence of the consumption of these drugs and the later development of cocaine addiction [28,35], it becomes indispensable to open up new pharmacological and therapeutic strategies to prevent their intake, focusing especially on adolescent subjects, who have proven to make up a very vulnerable population as regards suffering from drug-induced psychiatric alterations [11,55].

## Figures and Tables

**Table 1:** Effects of chronic adolescent WIN 55212-2 administration and cocaine withdrawal in adulthood on the elevated plus maze.

Measurement	Sal-Sal	Sal-Coca	WIN-Sal	WIN-Coca
Time OA (s)	97 ± 4.7	23 ± 9.9 **	92 ± 24	93 ± 28
% time OA	32 ± 1.6	8 ± 1 **	30 ± 2.5	31 ± 3
OA entries	23 ± 2.2	11 ± 1.7 *	21 ± 1.1	19 ± 2.1
% entries OA	51 ± 3	25 ± 4.1 **	51 ± 3.4	43 ± 2.3
Time CA (s)	130 ± 13	194 ± 11 **	123 ± 13	133 ± 13
CA entries	24 ± 2.6	34 ± 2.8 *	20 ± 2.6	24 ± 3.5
Total entries	47 ± 3.1	45 ± 2	41 ± 2.5	43 ± 3
Time center (s)	67 ± 9.2	78 ± 5.1	71 ± 4.9	64 ± 5.2

Across PND 34–47 (adolescence) mice (*n* = 10 per group) were pretreated with Sal or WIN (0.5 mg/kg given once daily). In adulthood (21 days after) they were treated with Coca in three daily injections separated by a 60-min interval according to the ensuing regime: 5 mg/kg on post-natal day (PND) 68 and 69, 15 mg/kg from PND 70 to 72, a 2-day abstinence period, and 25 mg/kg from PND 75 to 79. Control mice received the same injections of saline. Elevated plus maze was carried out on PND 82. OA: open arms, CA, closed arms. (* and ** *p* < 0.05 and 0.01 respectively, significantly different from the rest of the groups for each corresponding variable).

**Table 2:** Summary of the experimental design.

	Treatment					Behavioral Testing			
PND	34–47	48–67	68–69	70–72	75–79	80	81	82	83
Group	SAL	-	SAL	SAL	SAL				TST
	SAL		COCA 5	COCA 15	COCA 25	OF	PPI	OR	
	WIN		SAL	SAL	SAL	PPI	OR	EPM	
	WIN		COCA 5	COCA 15	COCA 25				

PND = Postnatal day; WIN = WIN 55212-2 (0.5 mg/kg) injected once a day during 14 consecutive days; COCA = cocaine given for three daily injections separated by a 60 min interval by the ensuing regime: 5 mg/kg on PND 68 and 69, 15 mg/kg on PND 70, 71 and 72, a 2 days abstinence period, and 25 mg/kg on PND 75, 76, 77, 78 and 79; SAL = saline (NaCl 0.9% *w*/*v*) given at the same treatment conditions as WIN and cocaine depending on the group. The experimental groups constituted were: Sal-Sal, WIN-Sal, Sal-Coca, and WIN-Coca. Behavioral testing began on PND 80. OF = open field, PPI = prepulse inhibition, OR = object recognition, EPM = elevated plus maze, TST = tail suspension test. For more details see the Materials and Methods section.
